# lncRNA MEG3 Suppresses the Progression of Ankylosis Spondylitis by Regulating the Let-7i/SOST Axis

**DOI:** 10.3389/fmolb.2020.00173

**Published:** 2020-07-24

**Authors:** Junjie Ma, Xiaohua Zhang, Hongxing Zhang, Hailong Chen

**Affiliations:** ^1^Third Department of Spine Surgery, Henan Luoyang Orthopedic Hospital (Henan Provincial Orthopedic Hospital), Luoyang, China; ^2^Department of Anesthesiology, Henan Luoyang Orthopedic Hospital (Henan Provincial Orthopedic Hospital), Luoyang, China

**Keywords:** AS, inflammation, bone formation, MEG3, let-7i, SOST

## Abstract

Ankylosis spondylitis (AS) is a disease mainly characterized by sacroiliac joint and spinal attachment point inflammation. Long non-coding RNA (lncRNA) plays a key role in the progression of many diseases. However, few studies have been conducted on the function of lncRNA maternally expressed gene 3 (MEG3) in AS. Quantitative real-time polymerase chain reaction (qRT-PCR) was used to measure the relative levels of MEG3, microRNA let-7i, sclerostin (SOST), and inflammatory cytokines. Dual-luciferase reporter assay, RNA immunoprecipitation (RIP) assay and biotin-labeled RNA pull-down assay were used to confirm the interaction between MEG3 and let-7i or let-7i and SOST. In addition, western blot (WB) analysis was performed to detect the protein levels of osteogenesis markers and SOST. The expression levels of MEG3 and SOST were decreased and let-7i was increased in AS patients. MEG3 could interact with let-7i in AS fibroblasts, and let-7i overexpression reversed the suppressive effect of MEG3 upregulation on the inflammation and bone formation of AS. Additionally, let-7i could target SOST, and SOST silencing reversed the inhibitory effect of let-7i inhibitor or MEG3 overexpression on the inflammation and bone formation of AS. Furthermore, SOST expression was positively regulated by MEG3, while was negatively regulated by let-7i. Our results revealed that lncRNA MEG3 promoted SOST expression to restrain the progression of AS by sponging let-7i, which provided a treatment target for AS.

## Introduction

Ankylosis spondylitis (AS) is a chronic systemic inflammatory disease that mainly affects the spine and sacroiliac joints, causing ankylosing and deformities (Ranganathan et al., 2017). The onset of AS is due to the occurrence of inflammatory responses and new bone formation based on synovial lesions, which eventually lead to ossification and rigidity of the entire joint (Smith, 2015; Watad et al., 2018). AS can cause patients with joint dysfunction, which causes great pain to patients and seriously affects patients’ daily lives and work (Kotsis et al., 2014). At present, the cause of AS has not yet been clarified, and genetic factors may be an important cause of AS (Brown and Wordsworth, 2017; Hanson and Brown, 2017). Therefore, fully understanding the molecular mechanisms that influence the inflammation and bone formation of AS has important guiding significance for the diagnosis and treatment of AS patients.

Both long non-coding RNA (lncRNA) and microRNA (miRNA) are common types of non-coding RNA (ncRNA) that have been studied extensively in recent decades (He et al., 2018). The hypothesis that lncRNA can be used as “miRNA sponge” has been confirmed in many studies, which has become an essential way to elucidate the molecular mechanism of lncRNA (Cui et al., 2017; Gao et al., 2019). Studies have shown that lncRNA has an important function in AS progression (Xu et al., 2019). For example, LINC00311 is upregulated in AS and can act as a biomarker for predicting AS treatment outcomes and recurrence (Zhong and Zhong, 2019), and low expression of TUG1 is thought to be associated with difficulty in treating AS patients (Lan et al., 2018). Maternally expressed gene 3 (MEG3) is a common tumor suppressor in cancers (Chen et al., 2020). Liu et al. (2019) suggested that MEG3 was lowly expressed in AS. However, studies on the role of MEG3 in the inflammation and bone formation of AS are still limited.

miRNA let-7i is considered as a biomarker for the diagnosis of AS patients (Reyes-Loyola et al., 2019), and many studies have confirmed that let-7i is significantly upregulated in AS patients (Li et al., 2016; Yang et al., 2019), but whether it regulates the inflammation and bone formation of AS remains unclear. Sclerostin (SOST) is a gene with significantly low expression in AS, and downregulation of SOST can promote osteoblast differentiation, bone formation and inflammation (Saad et al., 2012; Ma et al., 2019). Therefore, the study on SOST can help us better understand the role of its upstream regulator in the progression of AS.

In our experiments, we tested the expression levels of MEG3, let-7i and SOST in AS patients. Surprisingly, we not only verified that MEG3 and SOST were upregulated and let-7i was downregulated in AS, but also further found that the levels of let-7i and SOST were all correlated with the expression of MEG3. Through the predictions, we found that MEG3 could interact with let-7i, and let-7i could target SOST. Further functional tests confirmed the existence of the MEG3/let-7i/SOST axis in AS, which provided direct evidence for MEG3 to regulate AS progression.

## Materials and Methods

### Serum Samples Collection

Recruited 35 AS patients and 10 normal healthy peoples for routine physical examination from Henan Luoyang Orthopedic Hospital (Henan Provincial Orthopedic Hospital). Peripheral blood of AS patients and normal healthy peoples was collected and centrifuged at 2000 rpm/min for 10 min. The serum was collected and stored at −80°C for future use. AS capsule ligament tissues were collected for extracting primary AS fibroblasts. All AS patients and normal healthy peoples signed written informed content. Our study was approved by the Ethics Committee of Henan Luoyang Orthopedic Hospital (Henan Provincial Orthopedic Hospital).

### Primary AS Fibroblasts Isolation and Culture

The AS capsule ligament tissues were cut into pieces and digested with collagenase (Seebio Biotech, Shanghai, China) to obtain AS fibroblasts suspension. After filtration, the cell suspension was inoculated in a culture plate containing Dulbecco’s modified Eagle medium (DMEM; Hyclone, Logan, UT, United States) and cultured in a 5% CO_2_ humidified incubator at 37°C. The medium was replaced every 3 days. When the AS fibroblasts reached 80–90% fusion, the cells were inoculated into appropriate culture plates for subsequent testing. DMEM medium contained 10% fetal bovine serum (FBS; Invitrogen, Carlsbad, CA, United States) and 1% penicillin/streptomycin (Invitrogen).

### Quantitative Real-Time Polymerase Chain Reaction (qRT-PCR)

For the serum of AS patients and normal healthy peoples, BIOG cfDNA Easy Kit (Bio-generating Biotechnology, Changzhou, China) was used for extracting DNA from serums. For AS fibroblasts, total RNA was extracted using Trizol reagent (Invitrogen) and reverted to cDNA using the cDNA Synthesis Kit (Takara, Dalian, China). Then, SYBR Green (Thermo Fisher Scientific, Waltham, MA, United States) was used for measuring the expression of MEG3, SOST, interleukin-1β and 6 (IL-1β and IL-6), and tumor necrosis factor-α (TNF-α), and Tapman Universal PCR Master Mix (Thermo Fisher Scientific) was applied for detecting let-7i expression on PCR system. β-actin and U6 were used as the endogenous controls, and all data were normalized using the 2^–ΔΔCT^ method. All primers were listed as below: MEG3, F 5′-CTGCCCATCTACACCTCACG-3′, R 5′-CTCTCCGCCGTCTG CGCTAGGGGCT-3; let-7i, F 5′-GGGGTGAGGTAGTAGTT TG-3′, R 5′-TGCGTGTCGTGGAGTC-3′; SOST, F 5′-GAA CTAAGTTGCCGGGAGCT-3′, R 5′-CGGTAATCGGATGAGC TGCT-3′; IL-1β, F 5′-GAGTCTGCACAGTTCCCCAA-3′, R 5′-TGTCCCGACCATTGCTGTTT-3′; IL-6, F 5′-AACGATGA TGCACTTGCAGA-3′, R 5′-GAGCATTGGAAATTGGGGTA-3′; TNF-α, F 5′-ACACTCAGATCATCTTCTCAAAATTCG-3′, R 5′-GTGTGGGTGAGGAGCACGTAGT-3′; β-actin, F 5′-GAC CTCTATGCCAACACAGT-3′, R 5′-AGTACTTGCGCTCAGGA GGA-3′; U6, F 5′-AACGCTTCACGAATTTGCGT-3′, R 5′-CTC GCTTCGGCAGCACA-3′. The experiment was performed three biological replicates.

### Cell Transfection

All oligonucleotides and plasmids, including MEG3 overexpression plasmid (MEG3, 4.0 μg) and negative control (pcDNA, 4.0 μg), let-7i mimic and inhibitor (let-7i and in-let-7i) or negative controls (miR-con and in-miR-con, 50 nM), small interference RNA targeting MEG3 and SOST (si-MEG3 and si-SOST, 50 nM) or negative control (si-con, 50 nM) were synthesized from Ribobio (Guangzhou, China). Lipofectamine 3000 (Invitrogen) was used for AS fibroblasts transfection. The experiment was performed three biological replicates.

### Dual-Luciferase Reporter Assay

The sequences of MEG3 and SOST 3′UTR containing predicted and mutated binding sites with let-7i were sub-cloned into the psiCHECK-2 vectors (Promega, Madison, WI, United States) to generate MEG3 and SOST wild-type (WT) and mutant-type (MUT) vectors (MEG3-WT/MUT and SOST-WT/MUT). HEK 293T cells were obtained from Procell (Wuhan, China) and cultured in DMEM. Then, the vectors were co-transfected with let-7i, in-let-7i, miR-con or in-miR-con into HEK 293T cells using Lipofectamine 3000. The luciferase activity was measured using Dual-Luciferase Reporter Gene Detection System (Yeasen, Shanghai, China). The experiment was performed three biological replicates.

### RNA Immunoprecipitation (RIP) Assay

This assay was applied using RIP Kit (Millipore, Billerica, MA, United States). AS fibroblasts were transfected with miR-con or let-7i for 48 h. After that, AS fibroblasts were lysed and AS fibroblasts lysates were collected. Then, AS fibroblast lysates were incubated with magnetic beads conjugated with immunoglobulin G (IgG) or argonaute 2 (Ago2) antibody overnight at 4°C. QRT-PCR was performed to measure the enrichment of MEG3 and SOST in IgG or Ago2. The experiment was performed three biological replicates.

### Biotin-Labeled RNA Pull-Down Assay

Biotin-labeled let-7i-probe (Bio-let-7i-probe) or negative control probe (Bio-miR-con-probe; all from Sangon Biotech, Shanghai, China) was transfected into AS fibroblasts. After that, AS fibroblasts were lysed, and the AS fibroblast lysates were collected. Magnetic beads were incubated with AS fibroblast lysates overnight at 4°C. After that, Trizol reagent was used for extracting RNA, and the expression levels of MEG3, let-7i and SOST were determined by qRT-PCR. The experiment was performed three biological replicates.

### Western Blot (WB) Analysis

AS fibroblasts were lysed with RIPA buffer (Beyotime Shanghai, China) to extract total protein. After quantified using BCA Kit (Beyotime), equal amounts of proteins were separated with SDS-PAGE gel and transferred onto PVDF membranes (Millipore). Then, the membranes were blocked with 5% non-fat milk and incubated with antibodies against runt-related transcription factor 2 (RUNX2; 1:1,000, ab76956, Abcam, Cambridge, MA, United States), osteocalcin (OCN; 1:500, ab93876, Abcam), osteoprotegerin (OPG; 1:1,000, ab73400, Abcam), SOST (1:1,000, ab85799, Abcam) or β-actin (1:5,000, ab8227, Abcam) at 4°C overnight. After incubated with secondary antibodies against Goat Anti-Rabbit IgG (1:10,000, ab205718, Abcam) or Goat Anti-Mouse IgG (1:5,000, ab205719, Abcam), protein signals were visualized using enhanced chemiluminescence (ECL) reagent (Yeasen). The experiment was performed three biological replicates.

### Statistical Analysis

All data were analyzed using GraphPad Prism 6.0 (GraphPad Software, La Jolla, CA, United States) and presented as mean ± standard deviation. The comparisons analysis between different groups was carried out using Student’s *t*-test (one-tail, unpaired) or one-way analysis of variance. Pearson correlation analysis was used to analyze the correlations among MEG3, let-7i and SOST. The value of *P* < 0.05 was presented as a significant difference.

## Results

### The Expression of MEG3, Let-7i and SOST in AS Patients and Normal Healthy Peoples

**FIGURE 1:**
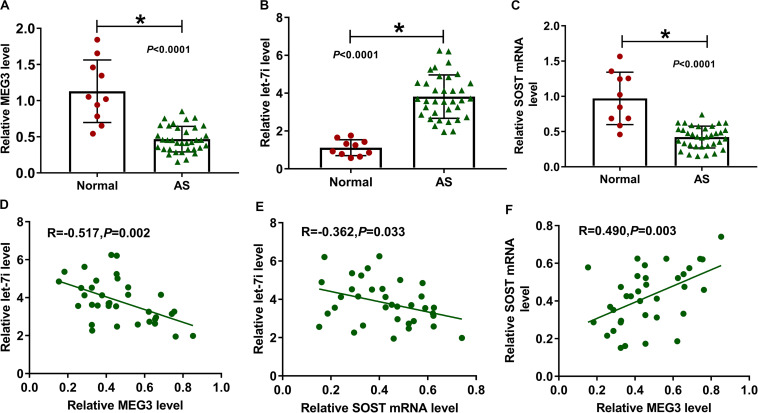
The expression of MEG3, let-7i and SOST in AS patients. **(A–C)** The expression levels of MEG3, let-7i and SOST in the serum of AS patients (AS, *n* = 35) and normal peoples (Normal, *n* = 10) were detected by qRT-PCR. **(D–F)** Pearson correlation analysis was used to analyze the correlations among MEG3, let-7i and SOST. All experiment required three biological replicates. *P < 0.05.

We first detected the expression of MEG3, let-7i and SOST in AS patients. Compared to normal healthy peoples, we found that the expression levels of MEG3 and SOST in the serum of AS patients were significantly decreased, while let-7i expression was markedly increased (Figures 1A–C). In addition, correlation analysis showed that the expression of let-7i was negatively correlated with the expression of MEG3 or SOST (Figures 1D,E), and MEG3 expression was positively correlated with SOST in AS patients (Figure 1F).

### MEG3 Directly Targeted Let-7i

**FIGURE 2:**
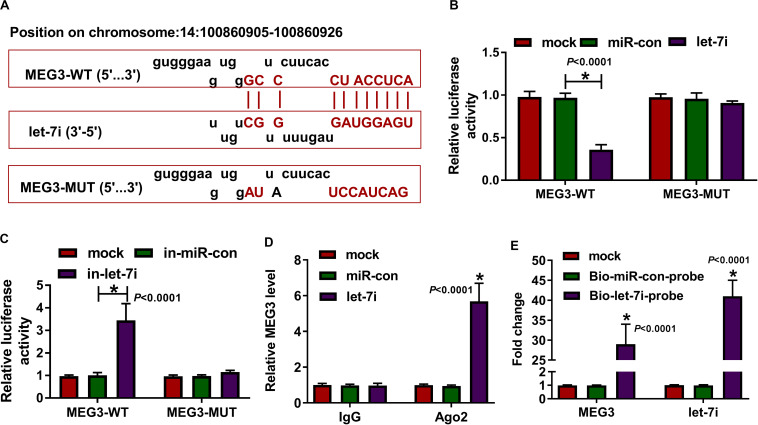
MEG3 directly targeted let-7i. **(A)** The fragments of MEG3-WT and MEG3-MUT were presented. Dual-luciferase reporter assay **(B,C)**, RIP assay **(D)** and biotin-labeled RNA pull-down assay **(E)** were used to verify the interaction between MEG3 and let-7i. All experiment required three biological replicates. *P < 0.05.

According the latest annotation provided by the Ensembl database, MEG3 had 50 isoforms. The LncBase Predicted v.2 software predicted that MEG3-201 (ENST00000398460.3) transcript combined with let-7i had the highest score (Figure 2A). Therefore, we constructed the MEG3-WT and MEG3-MUT vectors to perform the dual-luciferase reporter assay. The results revealed that let-7i overexpression remarkably inhibited the luciferase activity of MEG3-WT and its inhibitor obviously enhanced the luciferase activity of MEG3-WT, but neither had effect on MEG3-MUT (Figures 2B,C). In addition, by detecting the endogenous expression of let-7i and MEG3 after transfection of let-7i mimic or inhibitor, we found that let-7i upregulation and downregulation did not affect the expression of MEG3, which also indirectly confirmed that let-7i was a downstream of MEG3, and its abnormal expression had no effect on MEG3 expression (Supplementary Figures S1A,B). Meanwhile, the results of RIP assay indicated that high expression of let-7i could lead to a significant enrichment of MEG3 in Ago2 compared with IgG (Figure 2D). Additionally, pull-down assay also suggested that MEG3 and let-7i obtained a great enrichment in the Bio-let-7i-probe compared to the control group (Figure 2E). Our data presented that let-7i could be targeted by MEG3 in AS.

### Let-7i Overexpression Reversed the Inhibitory Effect of MEG3 Overexpression on the Inflammation and Bone Formation of AS

**FIGURE 3:**
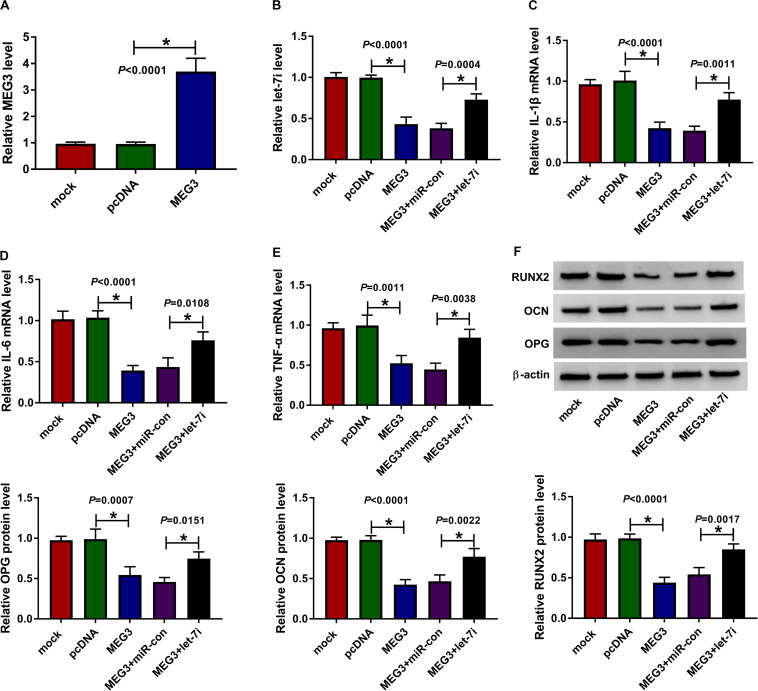
Effects of MEG3 and let-7i overexpression on AS progression. **(A)** MEG3 expression was detected using qRT-PCR to assess the transfection efficiency of MEG3 overexpression plasmid. AS fibroblasts were transfected with pcDNA, MEG3, MEG3 + miR-con or MEG3 + let-7i, respectively. **(B)** The expression of let-7i was measured by qRT-PCR to evaluate transfection efficiency. **(C–E)** The mRNA expression levels of IL-1β, IL-6 and TNF-α in AS fibroblasts were determined by qRT-PCR. **(F)** WB analysis was used to test the protein levels of RUNX2, OCN and OPG in AS fibroblasts. All experiment required three biological replicates. *P < 0.05.

For exploring the role of MEG3 and whether MEG3 regulated AS progression by targeting let-7i, we performed gain-functional and rescue-functional experiments. The increasing expression of MEG3 indicated that the transfection efficiency of MEG3 overexpression plasmid was good (Figure 3A). Through measuring the expression of let-7i, we found that MEG3 overexpression markedly inhibited let-7i expression, while let-7i mimic could reverse the suppression effect of MEG3 overexpression on let-7i expression (Figure 3B). The detection results of inflammatory cytokines expression suggested that upregulation of MEG3 could suppress the mRNA levels of IL-1β, IL-6 and TNF-α in AS fibroblasts, while this effect could be reversed by let-7i overexpression (Figures 3C–E). Moreover, we determined the protein levels of osteogenesis markers and the results suggested that let-7i mimic also could reverse the inhibition effect of MEG3 overexpression on the protein levels of RUNX2, OCN and OPG (Figure 3F). These results revealed that MEG3 restrained the inflammation and bone formation of AS by sponging let-7i.

### SOST Was a Target of Let-7i

**FIGURE 4:**
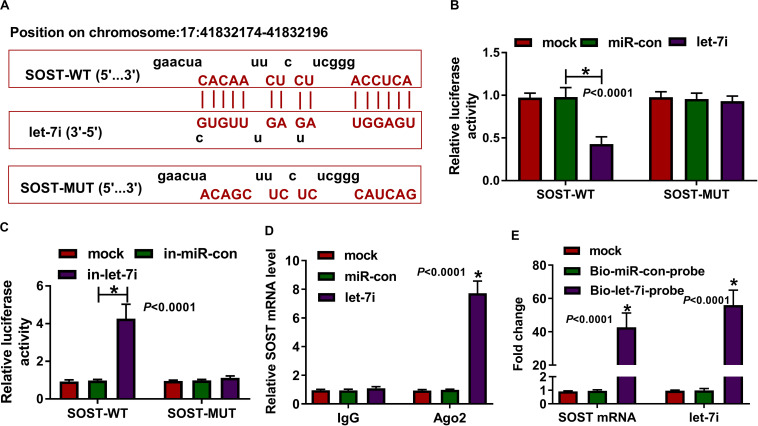
SOST was a target of let-7i. **(A)** The sequences of SOST-WT and SOST-MUT were shown. The interaction between SOST and let-7i was confirmed using dual-luciferase reporter assay **(B,C)**, RIP assay **(D)** and biotin-labeled RNA pull-down assay **(E)**. All experiment required three biological replicates. *P < 0.05.

In addition, the microT CDS tool predicted that there was a complementary binding site between SOST 3′UTR and let-7i (Figure 4A). Besides, dual-luciferase reporter assay results revealed that the luciferase activity of SOST-WT could be reduced by let-7i overexpression and promoted by let-7i inhibitor. However, the luciferase activity of SOST-MUT had not any changed (Figures 4B,C). Additionally, we also discovered that let-7i overexpression could markedly reduce the expression of SOST, while its inhibitor had an opposite effect, indicating that the expression of SOST was negatively regulated by let-7i (Supplementary Figures S1A,B). Furthermore, let-7i also could result in the expression of SOST significantly enriched in Ago2 (Figure 4D), and the results of pull-down assay indicated that the enrichments of SOST and let-7i were markedly increased in the Bio-let-7i-probe (Figure 4E). Hence, we suggested that let-7i could target SOST in AS.

### Silenced SOST Reversed the Suppressive Effect of Let-7a Inhibition on the Inflammation and Bone Formation of AS

**FIGURE 5:**
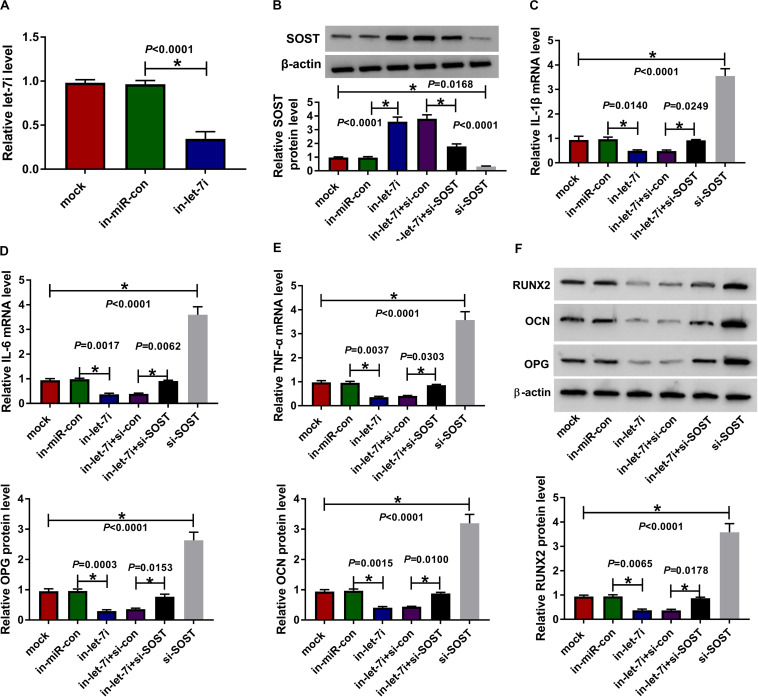
Effects of let-7a inhibitor and SOST silencing on AS progression. **(A)** The expression of let-7i was measured using qRT-PCR to evaluate the transfection efficiency of in-let-7i. AS fibroblasts were transfected with in-miR-con, in-let-7i, in-let-7i + si-con, let-7i + si-SOST or si-SOST, respectively. **(B)** The protein level of SOST was tested by WB analysis to confirm whether the transfection was successful. **(C–E)** QRT-PCR was performed to detect the mRNA expression levels of IL-1β, IL-6 and TNF-α in AS fibroblasts. **(F)** The protein levels of RUNX2, OCN and OPG in AS fibroblasts were determined using WB analysis. All experiment required three biological replicates. *P < 0.05.

To confirm the above results, we applied the rescue experiments using in-let-7i and si-SOST. QRT-PCR results indicated that in-let-7i remarkably repressed the expression of let-7i in AS fibroblasts, suggesting that the transfection efficiency of in-let-7i was excellent (Figure 5A). Besides, the detection results of SOST protein level revealed that si-SOST could markedly repress SOST expression and could reverse the promotion effect of let-7i inhibitor on SOST expression (Figure 5B). Through detecting the mRNA expression of inflammatory cytokines, we found that let-7i inhibitor restrained the mRNA expression of IL-1β, IL-6 and TNF-α in AS fibroblasts, while si-SOST had an opposite effect and could reverse the inhibition effect of let-7i inhibitor on the inflammation of AS fibroblasts (Figures 5C–E). In addition, SOST knockdown also inverted the suppression effect of let-7i inhibitor on the protein levels of RUNX2, OCN and OPG in AS fibroblasts (Figure 5F). Therefore, our data showed that let-7i targeted SOST to regulate the inflammation and bone formation of AS.

### MEG3 Regulated SOST Expression by Sponging Let-7i

**FIGURE 6:**
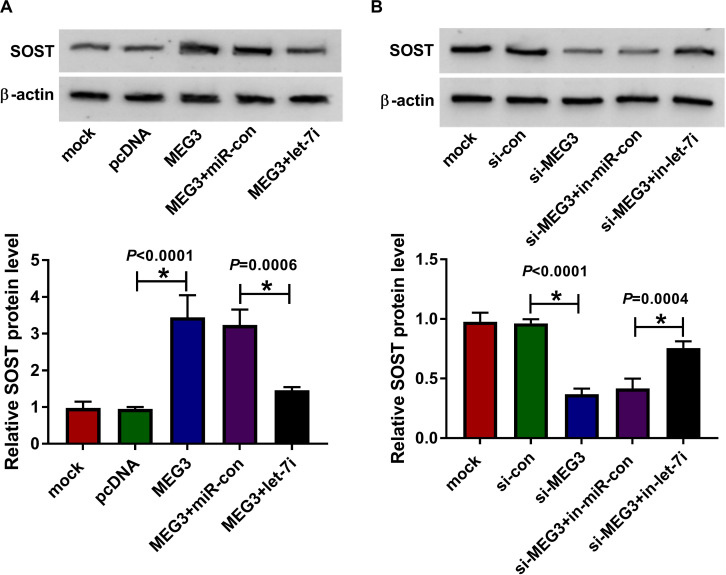
MEG3 and let-7i regulated SOST expression. **(A)** WB analysis was performed to measure the protein level of SOST to evaluate the effect of MEG3 overexpression and let-7i mimic on SOST expression. **(B)** The protein level of SOST was detected by WB analysis to assess the effect of MEG3 knockdown and let-7i inhibitor on SOST expression. All experiment required three biological replicates. *P < 0.05.

To clarify the regulatory effect of MEG3 on SOST, we measured the effect of MEG3 and let-7i expression on SOST expression. As shown in Figure 6A, MEG3 overexpression increased SOST protein level, while let-7i mimic reversed this promotion effect. On the contrary, MEG3 knockdown also suppressed SOST protein level, and this inhibitory effect could be recovered by let-7i inhibitor (Figure 6B). Therefore, our result concluded that MEG3 targeted let-7i to regulate SOST expression indirectly.

### SOST Knockdown Recovered the Suppressive Effect of MEG3 Overexpression on AS Progression

**FIGURE 7:**
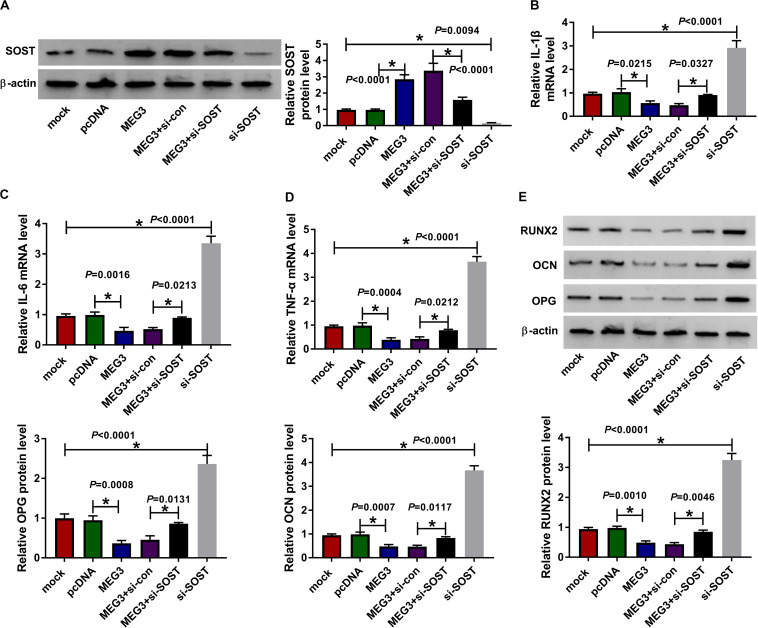
Effects of MEG3 overexpression and SOST knockdown on AS progression. AS fibroblasts were transfected with pcDNA, MEG3, MEG3 + si-con, MEG3 + si-SOST or si-SOST, respectively. **(A)** SOST protein level was measured using WB analysis to evaluate the transfection efficiency of MEG3 overexpression plasmid and si-SOST. **(B–D)** QRT-PCR was used to determine the mRNA expression levels of IL-1β, IL-6 and TNF-α in AS fibroblasts. **(E)** WB analysis was employed to detect the protein levels of RUNX2, OCN and OPG in AS fibroblasts. All experiment required three biological replicates. *P < 0.05.

For confirming MEG3 regulated AS progression by SOST, we co-transfected with MEG3 overexpression plasmid and si-SOST into AS fibroblasts. By measuring the protein level of SOST, we uncovered that si-SOST obviously inhibit SOST expression and could invert the promotion influence of MEG3 overexpression on SOST expression, suggesting that the transfection was successful (Figure 7A). Subsequently, we determined the inflammation and bone formation of AS fibroblasts. The results revealed that SOST knockdown could reverse the inhibitory effect of MEG3 overexpression on the mRNA expression of inflammatory cytokines and the protein levels of osteogenesis markers (Figures 7B–E). All data suggested that MEG3 suppressed AS progression by promoting SOST expression.

## Discussion

At present, there is no effective treatment for AS. Controlling inflammation and alleviating symptoms are the main treatment policy of AS (Dau et al., 2018). In the later period, combined with drug treatment and proper functional exercise can prevent the occurrence of spinal deformity to the greatest extent (van Weely et al., 2016). Therefore, it is necessary to explore new molecular targets to provide new pathways for the treatment of AS. In our study, we uncovered that MEG3 was downregulated in AS patients, which was consistent with the results of Liu et al. (2019). Additionally, a recent study indicated that MEG3 suppressed AS inflammation (Li et al., 2020). Here, our results showed good consistency with previous studies, and revealed that MEG3 could restrain the bone formation of AS to alleviate AS progression. In addition, our data suggested that MEG3 could sponge let-7i, and let-7i could target SOST. This is a new regulatory mechanism to regulate the progression of AS.

miRNA plays a key regulatory role in the development of AS, which has been confirmed in many studies. miR-17-5p is considered as an important target to promote the development of AS, and its expression is closely related to the heterotopic ossification of AS (Qin et al., 2019). The studies of Park et al. (2019) revealed that miR-451 had decreased expression in AS, and it could inhibit AS inflammation. For bone formation, miR-96 also could enhance osteoblast differentiation and bone formation to promote AS progression (Ma et al., 2019). Since let-7i was reported to be highly expressed in AS and its role was still unclear (Li et al., 2016; Yang et al., 2019), we focused on the role of let-7i in the progression of AS in this study. Additionally, the reversal effect of let-7i on MEG3 function also indicated that let-7i was involved in the regulation of MEG3 on the inflammation and bone formation of AS. The mechanism by which let-7i promoted the progression of AS was elucidated in our study.

Early studies have shown that SOST is a bone formation inhibitor secreted by bone cells and expressed specifically in bone cells (Winkler et al., 2003). And SOST has been used as a new drug target for anti-osteoporosis drug development (Weivoda et al., 2017). In AS, the low expression of SOST is considered as an important biomarker of AS progression, and its mechanism is similar to osteoporosis (Perrotta et al., 2018). Our results presented that the expression of SOST was lower in AS patients than in normal healthy peoples. Furthermore, we confirmed that MEG3 increased SOST expression to repress the inflammation and bone formation of AS through targeting let-7i, which confirmed the potential mechanism of SOST, as a vital target of anti-AS, regulated by the MEG3/let-7i pathway.

In summary, our results suggested that MEG3 played a negative role in AS progression, which could inhibit the inflammation and bone formation of AS by regulating the let-7i/SOST axis. Our findings enrich the molecular mechanism of MEG3 regulating the progression of AS and provide reliable and effective targets for the clinical treatment of AS.

## Data Availability Statement

The original contributions presented in the study are included in the article/Supplementary Material, further inquiries can be directed to the corresponding author.

## Ethics Statement

All AS patients and normal healthy peoples signed informed content. Our study was approved by the Ethics Committee of Luoyang Orthopedic-Traumatological Hospital of Henan Province (Henan Provincial Orthopedic Hospital).

## Author Contributions

JM performed the experiments and analyzed the data. HC wrote the manuscript. XZ designed the research, performed the experiments, and analyzed the data. HZ conceived and designed the research. All the authors contributed to the article and approved the submitted version.

## Conflict of Interest

The authors declare that the research was conducted in the absence of any commercial or financial relationships that could be construed as a potential conflict of interest.

## Supplementary Material

FIGURE S1 Effects of let-7i mimic and inhibitor on the expression of MEG3 and SOST. **(A)** AS fibroblasts were transfected with miR-con and let-7i. The expression levels of let-7i, MEG3 and SOST were determined by qRT-PCR. **(B)** AS fibroblasts were transfected with in-miR-con and in-let-7i. The expression levels of let-7i, MEG3 and SOST were measured using qRT-PCR. All experiment required three biological replicates. ^∗^*P* < 0.05.
