# Supplementary figures and images for: lncRNA MEG3 Suppresses the Progression of Ankylosis Spondylitis by Regulating the Let-7i/SOST Axis
Source: Front Mol Biosci. 2020 Jul 24;7:173. doi: 10.3389/fmolb.2020.00173 (PMC7393269; doi:10.3389/fmolb.2020.00173)

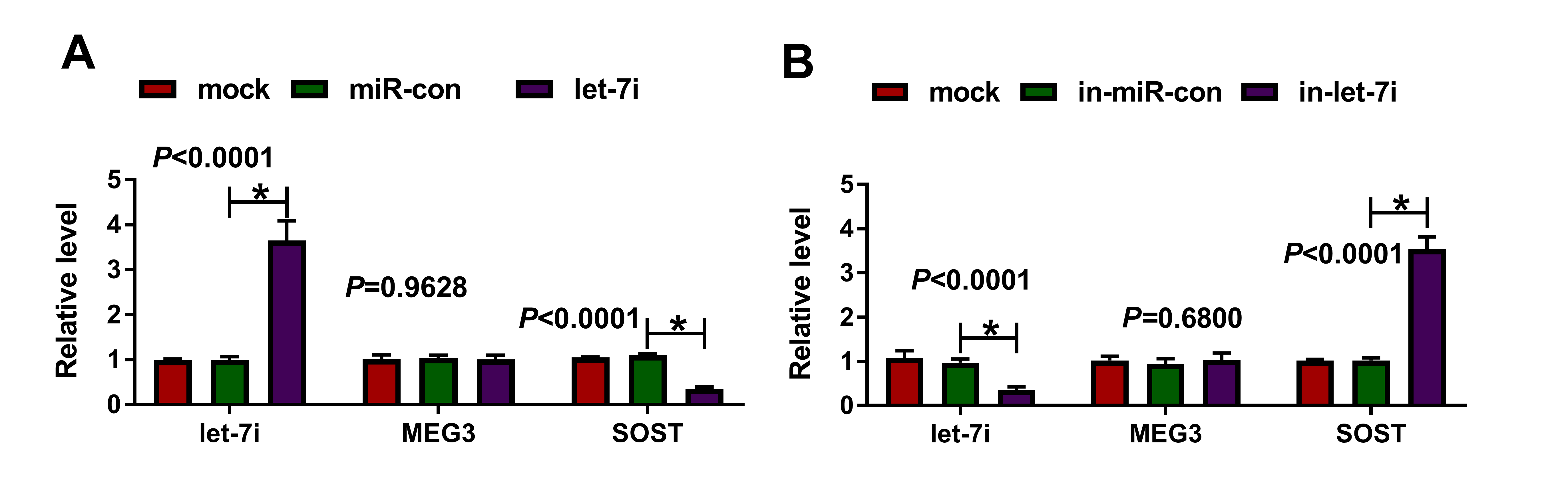

Supplement: FIGURE S1 — Effects of let-7i mimic and inhibitor on the expression of MEG3 and SOST. (A) AS fibroblasts were transfected with miR-con and let-7i. The expression levels of let-7i, MEG3 and SOST were determined by qRT-PCR. (B) AS fibroblasts were transfected with in-miR-con and in-let-7i. The expression levels of let-7i, MEG3 and SOST were measured using qRT-PCR. All experiment required three biological replicates. ∗P < 0.05. [file Image_1.TIF]
